# Nonodontogenic Cervical Necrotizing Fasciitis Caused by Sialadenitis

**DOI:** 10.1155/2016/9520516

**Published:** 2016-10-16

**Authors:** Alper Yenigun, Bayram Veyseller, Omer Vural, Orhan Ozturan

**Affiliations:** ^1^Department of Otorhinolaryngology, Faculty of Medicine, Bezmialem Vakif University, Fatih, Istanbul, Turkey; ^2^Department of Otorhinolaryngology, Faculty of Medicine, Acibadem University, Istanbul, Turkey

## Abstract

Necrotizing fasciitis is a rapidly progressive infectious disease of the soft tissue with high mortality and morbidity rates. Necrotizing fasciitis is occasionally located in the head and neck region and develops after odontogenic infections. Factors affecting treatment success rates are early diagnosis, appropriate antibiotic treatment, and surgical debridement. We present a necrotizing fasciitis case located in the neck region that developed after sialoadenitis. It is important to emphasize that necrotizing fasciitis to be seen in the neck region is very rare. Nonodontogenic necrotizing fasciitis is even more rare.

## 1. Introduction

The term necrotizing fasciitis was described initially by Wilson in the 1950s [1]. Necrotizing fasciitis is a rapid spreading disease of the soft tissue, which includes the superficial fascia and subcutaneous layer of tissue [2–4]. Necrotizing fasciitis is associated with some situations in which the immune system is compromised, including diabetes mellitus (DM), elderly, acute, or chronic renal disease, postpartum period, alcoholism, intravenous (IV) drug use, malnutrition, malignancy, peripheral vascular disease, and radiation exposure [5]. Necrotizing fasciitis diagnosis is based on certain clinical features that include fulminant progression, presence of grey-black necrotic area, and easy separation of the superficial layers of the underlying tissue [2, 6]. Necrotizing fasciitis can emerge from a local infection region after a minor trauma, which leads to the entrance of site of the infection. The exact etiology of one-third of the necrotizing fasciitis patients is not clear [2, 3, 6]. If necrotizing fasciitis is not diagnosed and treated early, it is potentially a fatal disease [4, 7, 8]. This situation is based on the absence of early clinical findings, rapid progression of the disease, and the delay of surgical intervention [9]. Therefore, experience in the diagnosis and treatment of necrotizing fasciitis is quite limited. Involvement of the head and neck region is quite rare for necrotizing fasciitis patients [2]. In this region there are two types of necrotizing fasciitis and they include cervical and craniofacial involvement [10]. Mortality rates of cervical necrotizing fasciitis range from 7% to 20% depending on the width of the cervical lesion [11]. This case report is presented because, unlike most of the others, this case of necrotizing fasciitis is nonodontogenic, sialoadenitis induced cervical necrotizing fasciitis.

## 2. Case Report

**Figure 1:**
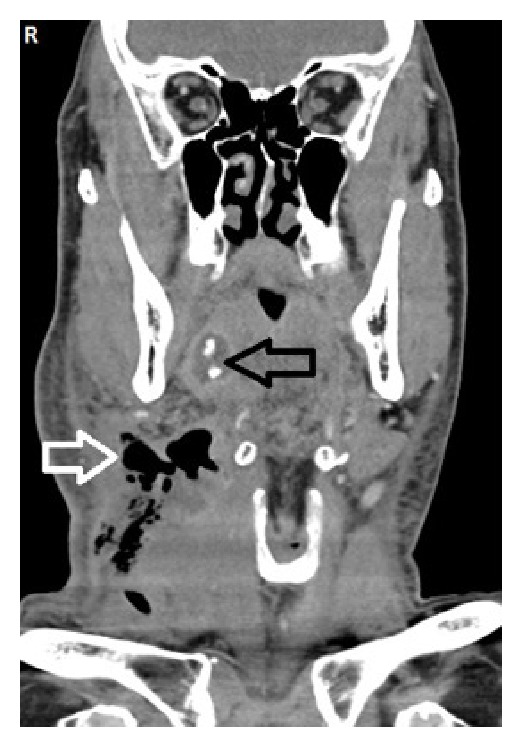
Coronal neck CT image showing cervical subcutaneous gas (white arrow) and right inflamed submandibular gland region with ductal stones (black arrow).

**Figure 2 fig2:**
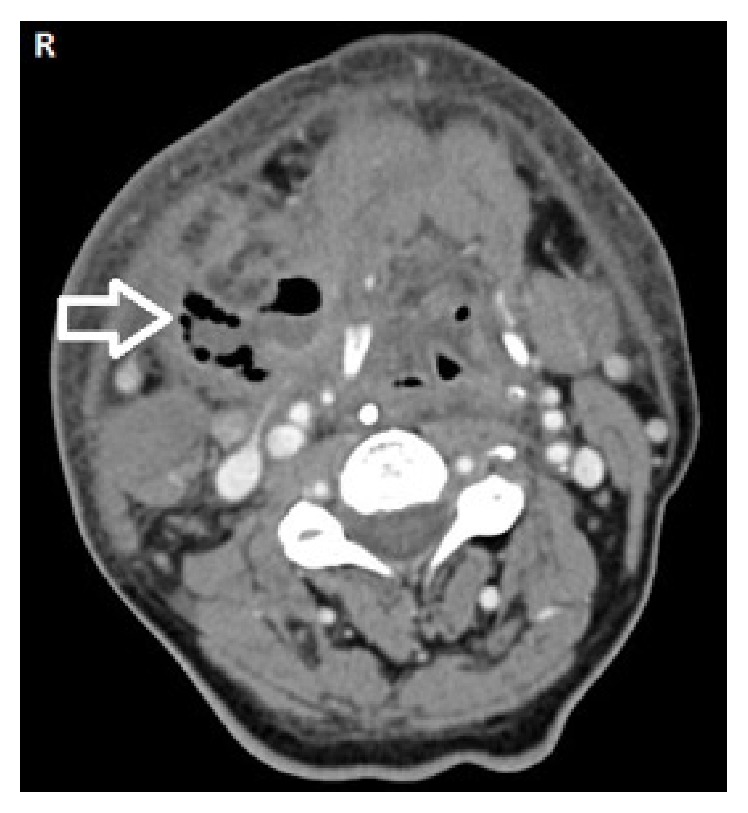
Axial neck CT image showing cervical subcutaneous gas (white arrow).

**Figure 3:**
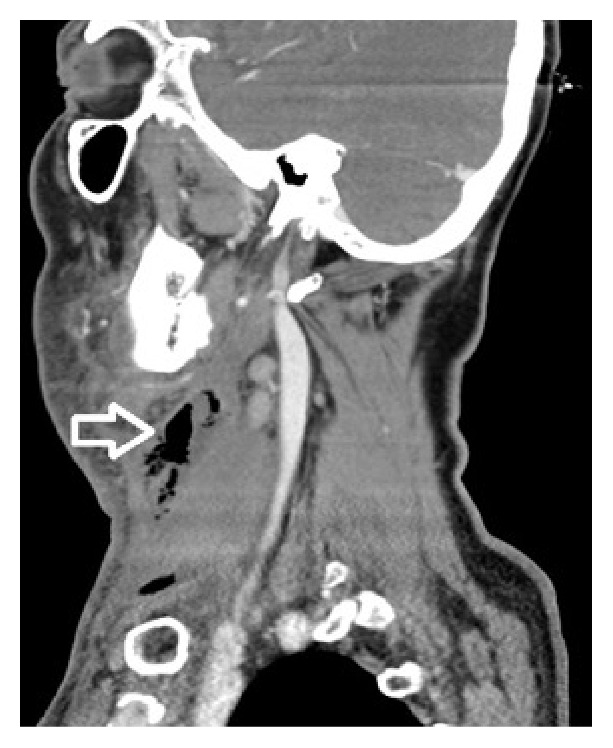
Sagittal neck CT image showing cervical subcutaneous gas (white arrow).

A 66-year-old male patient with complaints of fever, neck swelling, redness, and shortness of breath was admitted to the emergency department and on examination an edematous area was seen in the hyperemic region starting from the right submandibular area, spreading to the mastoid apex, right neck, and sternum. There was no known diabetes or immune deficiency in this case. Physical examination revealed a medium hard and crepitating swelling that was approximately 10 cm in dimension and was larger in the right side of the neck. On laryngoscope examination the vocal cords were edematous and had normal movement. Laboratory examination revealed a white blood cell (WBC) count of 24000/mm^3^ (neutrophils were prominent); sedimentation rate of 76 mm/h; C-reactive protein (CRP) of 31.9 mg/dL. Noticeably soft tissue edema was found in the neck ultrasound. Neck and thorax computed tomography (CT) showed right submandibular sialoadenitis and abscess induced by an intraductal stone. Widespread emphysema and edema were seen in the neck and mediastinum (Figures 1 [Fig fig2]–3). Considering these findings, necrotizing fasciitis was thought to be the diagnosis and the patient underwent wide cervical and thoracic debridement with exploration of the neck after the initiation of parenteral ampicillin 4*∗*1 gr for Gram-positive and Gram-negative bacteria, metronidazole 4*∗*0.5 gr for anaerobic organisms, and supportive therapy. The right submandibular gland was excised. After debridement the defect was left for secondary healing. In addition, 20 sessions of hyperbaric oxygen therapy were applied at 2.5 atmospheres (ATA) for 150 minutes. Mixed oropharyngeal flora prominent with anaerobic organisms was seen in the microbiologic examination of the pathology specimen. The patient did not develop any complications and the skin defects were covered completely with secondary healing during follow-up.

## 3. Discussion

Meleney described subcutaneous tissue necrosis caused by streptococcus in 1924 and used the term of “streptococcal gangrene” [12]. It is known that necrotizing fasciitis shows different behavior in each area. Necrotizing fasciitis is divided into two subgroups in the head and neck region. The first group involves the eyelids and scalp, with infection generally developing after trauma. The second group is seen rarely and shows involvement of the face and neck. Although the most common etiologic cause is dental infections, other reasons include trauma, peritonsillar abscess, osteoradionecrosis, infections of the tonsils or the pharynx, injury, foreign bodies, cervical adenitis, surgical wounds, tumors, and salivary glands [11, 13]. In addition to these, sialoadenitis with ductal stones may lead to necrotizing fasciitis by forming abscess [11]. In our case, sialoadenitis with ductal stones was also responsible for the necrotizing fasciitis causing abscess formation.

In this group of patients the disease extends to the chest wall and mediastinum at a rate of 65%, while 27% are fatal [7, 14–17]. Necrotizing fasciitis can affect people of all age groups regardless of gender or race [14]. Appropriate radiological examination and thorough assessment of the airway should be done without delay in order to determine the severity of the disease. Subcutaneous gas and abscess formation can be seen by CT [18]. Surgical treatment includes drainage and excision of all necrotic tissue with a wide fasciotomy incision and exploration of facial region [18]. Medical treatment required is broad spectrum antibiotics with fluid and electrolyte replacement [18]. Taking the anaerobic microorganisms into consideration, the hyperbaric oxygen treatment can be applied as support. In our case, after extensive surgical debridement, 20 sessions of hyperbaric oxygen therapy were given at 2.5 ATA for 150 minutes.

In conclusion, it is important to emphasize that necrotizing fasciitis to be seen in the neck region is very rare. Nonodontogenic necrotizing fasciitis is even more rare. It should be kept in mind that necrotizing fasciitis can be progressive and lead to deadly complications; the medical and surgical treatment should be applied without wasting time.

## References

[1] Wilson B. (1952). Necrotizing fasciitis. *The American Surgeon*.

[2] Fung V., Rajapakse Y., Longhi P. (2012). Periorbital necrotising fasciitis following cutaneous herpes zoster. *Journal of Plastic, Reconstructive & Aesthetic Surgery*.

[3] Benavides G., Blanco P., Pinedo R. (2003). Necrotizing fasciitis of the face: a report of one successfully treated case. *Otolaryngology—Head and Neck Surgery*.

[4] Dale R. A., Hoffman D. S., Crichton R. O., Johnson S. B. (2016). Necrotizing fasciitis of the head and neck: review of the literature and report of a case. *Special Care in Dentistry*.

[5] Safran D. B., Sullivan W. G. (2001). Necrotizing fasciitis of the chest wall. *The Annals of Thoracic Surgery*.

[6] Marioni G., Bottin R., Tregnaghi A., Boninsegna M., Staffieri A. (2003). Craniocervical necrotizing fasciitis secondary to parotid gland abscess. *Acta Oto-Laryngologica*.

[7] Umeda M., Minamikawa T., Komatsubara H., Shibuya Y., Yokoo S., Komori T. (2003). Necrotizing fasciitis caused by dental infection: a retrospective analysis of 9 cases and a review of the literature. *Oral Surgery, Oral Medicine, Oral Pathology, Oral Radiology, and Endodontics*.

[8] Liu Y. M., Chi C. Y., Ho M. W. (2005). Microbiology and factors affecting mortality in necrotizing fasciitis. *Journal of Microbiology, Immunology, and Infection*.

[9] Wong C.-H., Wang Y.-S. (2005). The diagnosis of necrotizing fasciitis. *Current Opinion in Infectious Diseases*.

[10] Zhang W.-J., Cai X.-Y., Yang C. (2010). Cervical necrotizing fasciitis due to methicillin-resistant *Staphylococcus aureus*: a case report. *International Journal of Oral and Maxillofacial Surgery*.

[11] Suárez A., Vicente M., Tomás J. A., Floría L. M., Delhom J., Baquero M. C. (2014). Cervical necrotizing fasciitis of nonodontogenic origin: case report and review of literature. *The American Journal of Emergency Medicine*.

[12] Djupesland P. G. (2000). Necrotizing fascitis of the head and neck—report of three cases and review of the literature. *Acta Oto-Laryngologica. Supplementum*.

[13] Feinerman I. L., Tan H. K. K., Roberson D. W., Malley R., Kenna M. A. (1999). Necrotizing fasciitis of the pharynx following adenotonsillectomy. *International Journal of Pediatric Otorhinolaryngology*.

[14] Sasindran V., Joseph A. (2011). Necrotizing fasciitis: an unusual presentation. *Indian Journal of Otolaryngology and Head & Neck Surgery*.

[15] Bulut M., Balci V., Akköse S., Armağan E. (2004). Fatal descending necrotising mediastinitis. *Emergency Medicine Journal*.

[16] Krenk L., Nielsen H. U., Christensen M. E. (2007). Necrotizing fasciitis in the head and neck region: an analysis of standard treatment effectiveness. *European Archives of Oto-Rhino-Laryngology*.

[17] Tung-Yiu W., Jehn-Shyun H., Ching-Hung C., Hung-An C. (2000). Cervical necrotizing fasciitis of odontogenic origin: a report of 11 cases. *Journal of Oral and Maxillofacial Surgery*.

[18] Scher R. L. (1998). Hyperbaric oxygen therapy for necrotizing cervical infections. *Advances in Oto-Rhino-Laryngology*.

